# European network for promoting the physical health of residents in psychiatric and social care facilities (HELPS): background, aims and methods

**DOI:** 10.1186/1471-2458-9-315

**Published:** 2009-08-28

**Authors:** Prisca Weiser, Thomas Becker, Carolin Losert, Köksal Alptekin, Loretta Berti, Lorenzo Burti, Alexandra Burton, Mojca Dernovsek, Eva Dragomirecka, Marion Freidl, Fabian Friedrich, Aneta Genova, Arunas Germanavicius, Ulaş Halis, John Henderson, Peter Hjorth, Taavi Lai, Jens Ivar Larsen, Katarzyna Lech, Ramona Lucas, Roxana Marginean, David McDaid, Maya Mladenova, Povl Munk-Jørgensen, Alexandru Paziuc, Petronela Paziuc, Stefan Priebe, Katarzyna Prot-Klinger, Johannes Wancata, Reinhold Kilian

**Affiliations:** 1 Department of Psychiatry and Psychotherapy II, Ulm University, Günzburg, Germany; 2 Dokuz Eylül Üniversitesi Tip Fakültesi Psykiyatri Anabilim Dali, Izmir, Turkey; 3 Department of Medicine and Public Health, Universitá degli Studi di Verona, Verona, Italy; 4 Queen Mary & Westfield College, London, UK; 5 Ozara Slovenia Life Quality National Association, Maribor, Slovenia; 6 Psychiatrické centrum Praha/Prague Psychiatric Centre, Praha/Prague, Czech Republic; 7 Department of Psychiatry and Psychotherapy, Medical University of Vienna, Vienna, Austria; 8 Fondatzia za Choveshki Otnoshenia, Sofia, Bulgaria; 9 Vilnius University, Vilnius, Lithuania; 10 Mental Health Europe-Sante Mentale Europe, Brussels, Belgium; 11 Unit for Psychiatric Research, Åalborg Psychiatric Hospital, Aarhus University, Åalborg, Denmark; 12 Department of Public Health, University of Tartu, Tartu, Estonia; 13 Institute of Psychiatry and Neurology, Warsaw, Poland; 14 Fundació Institut Català de l'Envelliment, Universitat Autonoma de Barcelona, Barcelona, Spain; 15 Campulung Moldovenesc Psychiatric Hospital, Campulung Moldovenesc, Romania; 16 London School of Economics and Political Science, London, UK

## Abstract

**Background:**

People with mental disorders have a higher prevalence of physical illnesses and reduced life expectancy as compared with the general population. However, there is a lack of knowledge across Europe concerning interventions that aim at reducing somatic morbidity and excess mortality by promoting behaviour-based and/or environment-based interventions.

**Methods and design:**

HELPS is an interdisciplinary European network that aims at (i) gathering relevant knowledge on physical illness in people with mental illness, (ii) identifying health promotion initiatives in European countries that meet country-specific needs, and (iii) at identifying best practice across Europe. Criteria for best practice will include evidence on the efficacy of physical health interventions and of their effectiveness in routine care, cost implications and feasibility for adaptation and implementation of interventions across different settings in Europe. HELPS will develop and implement a "physical health promotion toolkit". The toolkit will provide information to empower residents and staff to identify the most relevant risk factors in their specific context and to select the most appropriate action out of a range of defined health promoting interventions. The key methods are (a) stakeholder analysis, (b) international literature reviews, (c) Delphi rounds with experts from participating centres, and (d) focus groups with staff and residents of mental health care facilities.

Meanwhile a multi-disciplinary network consisting of 15 European countries has been established and took up the work. As one main result of the project they expect that a widespread use of the HELPS toolkit could have a significant positive effect on the physical health status of residents of mental health and social care facilities, as well as to hold resonance for community dwelling people with mental health problems.

**Discussion:**

A general strategy on health promotion for people with mental disorders must take into account behavioural, environmental and iatrogenic health risks. A European health promotion toolkit needs to consider heterogeneity of mental disorders, the multitude of physical health problems, health-relevant behaviour, health-related attitudes, health-relevant living conditions, and resource levels in mental health and social care facilities.

## Background

**Figure 1:**
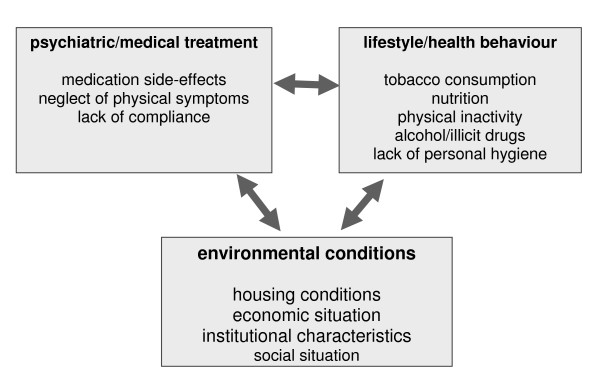
**Potential causes of physical comorbidity in people with severe mental illness**.

A large number of studies and reviews from different countries have revealed that people with mental disorders have higher rates of physical illness than the general population, and that their risk of premature death due to physical illness is increased [1-9]. Higher incidence and prevalence rates for cardiovascular disease (e.g. ischemic heart disease, cardiac arrhythmias, myocardial infarction) [1,10], diabetes mellitus (associated with obesity, impaired glucose tolerance and insulin resistance) [11,12], several types of cancer and infectious (e.g. HIV/AIDS) as well as gastrointestinal diseases translate into reduced life expectancy [1,9,13-18]. Several studies have examined physical comorbidity in patients with a diagnosis of schizophrenia [11,18-23]. Overall mortality in this target group is double that of the general population [2]. Thus, schizophrenia has been called a "life-shortening disease" [24]. Causes of unnatural death such as suicide and accidents account for only part of this increase in mortality rate; a substantial proportion (59%) is due to natural causes including physical illness [25,26]. There are suggestions that almost 50% of patients with schizophrenia may suffer from comorbid physical conditions [19,23]. Diabetes mellitus, cardiovascular disease, hypertension, osteoporosis, respiratory disease, and obesity are among the most common conditions [19-22] in this group. Most studies have focused on schizophrenia, but excess mortality due to physical illness is more widespread and has also been found in other diagnostic groups, e.g. affective and anxiety disorder [27-37]. Figure 1 shows you several potential causes of physical comorbidity in people with severe mental illness.

### Behavioural-related causes of physical co-morbidity

The higher prevalence of somatic conditions in people with mental illness may be related with the mental disorder itself and/or unhealthy lifestyle, such as excessive smoking, poor nutrition, physical inactivity, insufficient hygiene, dental and sexual health behaviour and hazardous/risk-taking behaviour [14,38-40]. Increased prevalence rates for smoking were found among people with schizophrenia, bipolar and depressive disorder, neurotic and somatoform disorder [41]. A meta-analysis of 42 studies from 22 countries reported a prevalence of smoking in people with schizophrenia to be about seven times the general population average for males and three times higher for females [38]. This leads to a higher rate of nicotine-related diseases [1,40,42] and is regarded by several authors as one of the major causes of high somatic morbidity and also mortality in this patient group [40,43-45]. The odds of hazardous alcohol consumption were three times higher among patients with depression and doubled for those with schizophrenia [41] although alcohol use can exacerbate psychotic symptoms and medication side-effects, e.g. extra pyramidal symptoms [46]. Illicit drug use was found to be much higher in patients with schizophrenia, bipolar disorder, depression, neurotic and somatoform disorder [41,44,46]. Unhealthy food habits were found to be increased across diagnostic groups [41], which included higher proportions of saturated fats, lower consumption rates of fresh vegetables and fruit and lower fibre content compared with the general population [26,40,44,45]. Only a very few studies examined physical activity and reported lower levels of regular exercise among people with schizophrenia [40]. Higher rates of unsafe sex practices with multiple partners and sexual exploitation among people with mental disorders (particularly schizophrenia) living in group care homes, hostels or on the street constitute a risk factor for HIV and AIDS [1,46]. In general, studies on unhealthy lifestyle habits and health behaviour of people with mental illness are for the most part limited to people with schizophrenia and only little is known about the extent of unhealthy lifestyle in other diagnostic groups [38,44].

### Environmental causes of physical co-morbidity

Health behaviour is influenced by personal features that interact with social context variables (e.g. social support, norms and beliefs) and physical factors (e.g. weather, climate, means of transport, buildings, nutrition and recreational facilities) [26,47,48]. Furthermore, the living environment can be a direct determinant through environmental constraints and supports, such as physical activity [49]. Research on physical activity and neighbourhood environments indicates that people are more physically active in neighbourhoods with leisure opportunities [50,51]. A recent study by Karlin and Zeiss [52] revealed that sunlight in patient rooms may promote recovery of patients with severe depression, that exposure to and views of nature can lead to reduced psychological distress and fatigue and facilitate recovery, and that big and low windows may improve sensory abilities and reduce delirium as well as paranoia. Single or non-dormitory-style resident rooms enhance privacy and autonomy and may promote participation in treatment activities. Architecture and building standards have direct effects on residents' physical health; they shape behavioural opportunities [26,53], e.g. individual hygiene (bathrooms), physical activities (gymnasiums, opportunities for sport) and risk behaviour opportunities (smoking rooms, proximity of pubs). Organisational aspects, e.g. routine for daily hygiene and preparation of meals, availability of energy-dense foodstuffs, regulations and rules e.g. smoking bans also have effects on residents' physical health.

### Professional care-related causes of physical co-morbidity

There are several health system-related factors, including professional care-related factors. For example, bipolar patients with a comorbid physical condition often receive inadequate treatment for their physical condition [33]. Psychiatrists are highly specialised doctors who may, in common with other specialists, have little or distant experience of general practice. Opportunities for the early detection and treatment of physical illness in mentally ill may therefore be missed [43]. Psychiatric services, with the encouragement of policy makers, have tended to concentrate on the direct consequences of psychotic illness, for example suicide and dangerousness, rather than the indirect effects such illness have on physical health [43]. Additionally, there is evidence that patients with schizophrenia have lower rates of utilisation of physical health care services and the quality of the physical care they receive is worse as compared with the general population [54]. Furthermore there is significant empirical evidence that the physical health of people with mental illness is often disregarded by mental or general health care institutions [1,55]. At present, neurobiological, psychosocial and treatment-related factors are assumed to be responsible for the high prevalence rates of smoking, alcohol and substance abuse among people with mental illness [56-62]. In spite of the growing evidence of co-occurrence of mental and somatic disorder, the nature of relationships between the two groups of conditions is multiplex due to the multitude of mechanisms of action and interaction.

### The need for prevention programmes

Knowledge on the causes of increased somatic morbidity and mortality is fragmentary, and standardised screening instruments for somatic health risks in this population are not available in routine practice. There is a shortage of systematic efforts or programmes for the prevention of physical illness in people with mental disorder [63]. On the other hand, physical health promotion programmes for people with mental health needs have been successfully applied in diverse settings, e.g. local communities [64], schools [65], prisons [66] and medical care settings [67]. The majority of these intervention programmes have focused on smoking cessation [68,69], and there are some programmes for the improvement of dietary behaviour and physical exercise [63,70]. Many of these programmes have been initiated by pharmaceutical companies, and they are related to the issue of antipsychotic-induced weight gain. Most intervention programmes aim at changing patient behaviour, whereas they tend to neglect the role of (unhealthy) environmental living conditions [38,44]. Most health promoting intervention programmes were developed and tested in the USA or UK, and this also limits our knowledge regarding feasibility in other countries. Thus, there is a striking lack of programmes targeting unhealthy lifestyle factors related to poor physical health in people with mental health problems [39]. Current national surveys and cohorts cannot provide sufficient information to improve physical health in people with mental illness, and there appears to be a lack of resources dedicated to dealing with somatic comorbidity.

**Figure 2:**
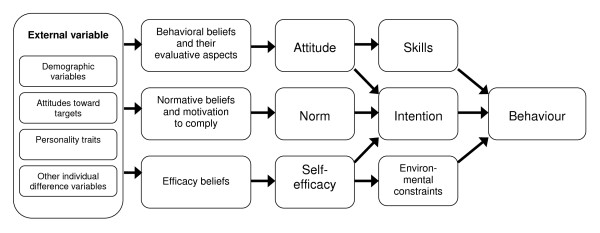
**The role of health-related attitudes in the process of health behaviour**. Glanz et al., 2002.

It must not be forgotten that health activities and interventions aimed at reducing unhealthy lifestyles may actually have negative effects on the subjective quality of life of people with mental health problems if these actions are perceived as restrictive (e.g. smoking bans, restrictions of sexual contacts) or as paternalistic (e.g. change of nutrition habits, urge for physical activity). Additionally, smoking can diminish negative symptoms in schizophrenia and medication side-effects, e.g. extra pyramidal symptoms, and it may be perceived as a method to improve concentration, reduce boredom, negative feelings or anxiety [56-58,60,71-74]. Nicotine consumption, alcohol, and illicit drug use may also be perceived as improving social interaction, mood and coping with distress [57,59,75]. Thus, the unhealthy lifestyle and health behaviours in people with mental disorder are probably related to interactions of biological and environmental factors, as well as to normative beliefs, attitudes, and behavioural beliefs, which are personal beliefs about the role of behavior in the process of health and illness (self-efficacy). The integrative model (figure 2) by Glanz and colleagues [76] represents the linkage of these factors and how they impact on behaviour. This complexity has to be taken into account in planning intervention programmes.

### European perspective

The diversity of the European region is evident in psychiatric care (as in many other fields). Psychiatric facilities in Eastern European countries have to provide mental health care at lower resource levels than in Western Europe; there is a greater reliance on long-stay institutions yet many are still to catch up with international developments. On average, the mental health budget is 5.6% of the total health budget across Europe, but the proportion varies from less than 1% in some parts of Eastern Europe to about 12% in some Western European countries [41]. The availability of services and coverage by public health systems varies. Psychiatric hospitals in some Eastern European countries may lack the equipment and services to recognise and treat physical ill health in people with mental health needs and primary care or internal medicine input may not be readily available [77]. Eastern European countries have to cope with low healthcare funding levels, and out-patient facilities may not be within easy reach. Community care may not be available [77-80], and the workload due to mental disorders, including substance abuse, may be increasing in a context of social instability [77]. While countries in the eastern half of the European Union may be catching up with their western European counterparts mental health appears to still be a recipient of "second-class treatment".

National and international pharmaceutical companies that provide modern psychiatric drugs across Europe influence the field of mental health in rich and poor countries alike. Thus it can be considered a success that innovative drugs for treating mental disorders are available in some low resource countries in Central and Eastern Europe. But providing psychiatric drugs can make the introduction of psychosocial therapies more difficult in countries with a low level of healthcare resources. For example, while the Lithuanian health care system spends an average of two to three million euros on modern psychiatric drugs, funding for the psychiatric treatment of children and young people in the secondary and tertiary sectors is limited to around 1 million euros [81].

### Empowerment aspect

The individual should not be considered a passive object in any prevention strategy but instead take an active part in the health promotion process [82]. The setting approach to health promotion should focus on strengthening individual abilities and resources of people with mental illness to empower them to "achieve their fullest health potential" by "making healthy choices" (World Health Organization). According to the Ottawa Charta 1996 health promotion programmes should be based on the concept of empowerment to help individuals increase the degree of control over their health-related living conditions and quality of life. There is evidence based on multi-level research designs that empowering initiatives can improve health outcomes [83]. Service user empowerment has developed as a proactive partnership and self-care strategy to improve health outcomes and quality of life among the chronically ill [84,85]. Wallerstein defines empowerment as "a social-action process that promotes participation of people, or organizations, and communities towards the goals of increased individual and community control, political efficacy, improved quality of life, and social justice"[86]. Thus empowerment is a complex strategy in complex environments: effective empowerment strategies may depend as much on the agency and leadership of the people involved as on the context in which they are implemented [83].

### Need for the project and purpose of HELPS

The obvious neglect of the physical health status of people with mental illness in general, and of residents in psychiatric and social health care facilities in particular, is incompatible with the protection of human rights and dignity. Poor physical health in this population must begin to receive the attention that it deserves, particularly in view of the substantial numbers of individuals who spend time in residential facility settings across Europe. There is a need for a physical health promotion toolkit that can be implemented not only in the community at large but also in mental health and social care facilities across the European Union. The toolkit must comprise an empowerment strategy which enables professionals and residents of mental health care facilities to routinely identify relevant health problems, explore the main causes, and then choose and implement appropriate health promotion programmes suitable to the range of facilities.

## Methods and design

HELPS is a European multi centre project with a project period from 2008 to 2010, funded by the European Union, in the framework of the Public Health Programme. The 15 countries with centres participating in the project cover about 80% of the inhabitants of the European Union member states and about 94% of the inhabitants of the candidate countries from all geographical regions of the enlarged Europe. Undertaking this project within a consortium allows the collection of representative information on health-related conditions in mental health care facilities, including the physical health status of residents and health-related attitudes of residents and staff members across Europe. The broad geographical coverage in this project is of particular importance because the great diversity in economic circumstances across the participating countries is expected to have a significant impact on health-related conditions in mental health care facilities. As such HELPS is an interdisciplinary European network developing health promotion initiatives that meet country-specific needs. It will identify best practice (evidence of the efficacy of physical health interventions and of their effectiveness in routine care, cost implications issues and feasibility for adaptation and implementation of interventions) across different settings in Europe.

### Project objectives and aims

The overarching objective of HELPS is the development of a framework of empowerment focusing on the promotion of healthy living conditions in psychiatric care facilities and the improvement of the physical health status of residents of psychiatric and social care facilities in the European Region.

For this purpose it is intended to put together a selection of validated screening instruments and effective and efficient health promotion programmes as an up-to-date and feasible toolkit to facilitate the treatment of physical illness in people with mental health needs that is suitable for application in a wide range of mental health care facilities across Europe. The HELPS toolkit will be embedded in an empowerment framework for health promotion in mental health care. Users should be qualified to choose and implement suitable health promotion programmes at the individual, organisational and environmental level. Staff members and residents in these facilities should be enabled to identify routinely the most relevant physical health problems, to detect adverse and protective health-related lifestyles and attitudes, to discover relevant facility characteristics, and HELPS should increase staff members' and residents' control over their living conditions and their confidence in the effect of their own actions to improve health status. To assess the validity and feasibility of applying the health promotion toolkit, a pilot application phase will be carried out in at least one psychiatric or social care facility in each of the participating countries. Experiences from this pilot phase will be used to improve parts of the toolkit which are not suitable for routine application.

It is planned to translate the HELPS toolkit into the following European languages: English, Bulgarian, Danish, Czech, Estonian, French, German, Italian, Lithuanian, Polish, Romanian, Slovenian, Spanish, and Turkish. This would cover the languages of about 80% of the citizens of EU member states and about 96% of the citizens of candidate countries. A standardised translation procedure should facilitate adaptation to further languages. Thus we expect to enable access not only to the participating centres but to all individuals or facilities who are interested in the health promotion of people with mental illness. The HELPS toolkit shall be accessible via the internet and as a CD ROM. We hope that media campaigns will be a helpful way for dissemination, application, and implementation of the toolkit. It will be complemented by a training manual using multimedia presentations to introduce key targets of the HELPS toolkit and describe its component parts. Furthermore it is projected to offer workshops on the use of the paper-pencil and computer versions of assessment instruments. This practical training should enable participants to pass on the skills required to handle the toolkit in peer groups and, in this way, they could become multipliers in dissemination of the toolkit.

## Methods

The key methods used in this project are: (a) international literature reviews, (b) stakeholder analysis, (c) Delphi exercises, and (d) focus groups:

(a) ***International literature reviews ***(ILR) will be used to collect fragmented knowledge on the prevalence of physical illness in people with mental illness and information on standardised methods for the assessment of physical health status, health-relevant lifestyle, and health related attitudes among people with mental disorder to get a comprehensive picture of the international knowledge and the international state of the art of measurement in this field. ILR will also be used to identify effective and cost-effective health promotion programmes appropriate for routine use in mental health and social care facilities. In addition to searching electronic databases, HELPS partners will also search the "grey literature" to identify other useful sources of information on these topics.

(b) Heterogeneity in different respects (e.g. policy, economic situation, cultural norms) between and within the participating countries is evident. Therefore, extensive consultation with relevant stakeholders will be undertaken. ***Stakeholder analysis ***[87] will be conducted in each country to identify relevant stakeholders for the HELPS project and to determine the best ways they can be involved in the project. The stakeholder analysis has the intention of developing collaborative links between stakeholders and project partners. Stakeholders will act as advisors, name the relevant multipliers for the dissemination of project results and the toolkit, and they will ensure the development successful project outcomes.

(c) Participants of ***Delphi exercises ***will be identified through stakeholder analysis, and these will be selected on the basis of their expertise, rated through publications or positions held. Delphi exercises will be conducted to identify available data (1) on the prevalence and causes of physical health problems in residents of mental health care facilities, (2) on the adequacy of assessment methods, (3) on the availability of health promotion programmes, and on the extent of their implementation in mental health care facilities.

(d) ***Focus groups ***[88-90] will be conducted in acute and long-stay facilities in each participating country in order to capture the social, economic, and cultural diversity of the enlarging European Union. The aim is to gather information on residents' and staff members' subjective perception of physical health problems, their thoughts and beliefs on the causes of the physical health risks, and their acceptance and ideas regarding physical illness prevention strategies and interventions programmes for physical health promotion. Since the focus group method is an explorative qualitative research technique, no formal power calculation can be made to determine sample size. Instead, the variety and the number of focus groups to conduct will be determined on the basis of the theoretical sampling model [91,92], and by the level of segmentation of the study population. Once the criteria for segmentation have been identified (in our case: "professionals" and "patients/residents"), it is usual practice to conduct at least two focus groups within each sub-group of the study population [93-97]. Initially, all centres will start with conducting one focus group with residents and one focus group with staff members. After the data of the first focus groups are analyzed researchers in the centres will decide whether a stage of theoretical saturation is reached or whether additional groups are necessary.

Inclusion criteria for residents are: (a)participants have to be 18 years old or older, and (b) they need to have a psychiatric diagnosis. Resident exclusion criteria are: (a) substance abuse as primary diagnosis; (b) the presence of a moderate or severe intellectual disability or organic mental disorder (such as Alzheimer's Disease); (c) a current treatment by forensic psychiatric services; (d) the insufficient command of the national language in order to take part in the focus group; and (e) a lack of capacity to give valid consent.

The HELPS Coordinating Centre (Ulm University) requested and received ethics approval for the project from the "Ethikkommission der Universität Ulm", Germany. In addition, the following centres requested and received ethics approval from their appropriate facility review board and/or ethics committee: Austria: Ethik-Kommission der Medizinischen Universität Wien und des Allgemeinen Krankenhauses der Stadt Wien; Czech Republic: Eticka komise Psychiatrickeho centra Praha; Denmark: Den Videnskabsetiske Komite for Region Nordjylland; Estonia: Ethics Review Committee (ERC) on Human Research of the University of Tartu; Italy: Comitato Etico Provinciale per la sperimentazione clinica di Verona; Lithuania: Vilniaus Regioninis Biomedicininiu Tyrimu Etikos Komitetas; Poland: Instytut Psychiatrii i Neurologii komisja bioetyczna; Slovenia: Komisija Republike Slovenije za Medicinsko Etiko; Spain: Comision de Etica en la Experimentacion Animal y Humana (CEEAH); Turkey: Ethical Committee for Clinical and Labaratuar Research of Dokuz Eylül University School of Medicine; United Kingdom: East London and the City Local Research Ethics committee 2.

## Discussion

Improving the physical health of people with mental illness is a complex issue. Knowledge and awareness of risk factors relevant to physical health is required in order to develop behavioural or environmental prevention programmes. A European health promotion programme must take into consideration that there is substantial variation across countries regarding the use of residential care facilities, the extent to which they consider physical health problems, health-relevant behaviour, health-related attitudes of service users/residents, health-relevant living conditions and resource levels in residential mental health care facilities.

A widespread use of the "HELPS health promotion toolkit" is expected to have a significant positive effect on the physical health status of residents of mental health and social care facilities, as well as to hold resonance for community dwelling people with mental health problems. In addition, dissemination of the toolkit will become one element of a European strategy to improve living conditions in these facilities. Furthermore, it is expected that the HELPS toolkit will be perceived by staff members and residents of mental health care facilities as an opportunity to increase control and enhance their quality of life.

However, it should be recognised that a substantial proportion of the excess physical morbidity may be not address by tackling these factors. Some of the risks of physical ill health in people with mental illness are likely to be inherent. Changes in the immune system and hormonal unbalance are potential risk factors. According to the Mental Health Declaration and the Mental Health Action Plan for Europe , agreed upon in Helsinki in conditions among people with mental illness in Europe is now a priority of European health policy. The promotion of healthy living conditions and the prevention of physical comorbidity are preconditions for maintaining the dignity and the human rights of residents of mental health care facilities. In summary, we expect that these project outcomes will also make an important contribution to maintaining and enhancing the dignity, human rights and general health of residents of mental health care facilities in Europe.

## Conclusion

Due to the great variance in health-relevant characteristics of mental health care facilities in participating countries, the provision of a uniform health promotion programme for all facilities would not be appropriate. On the other hand, the mere provision of a number of health promotion programmes to choose from would be insufficient because the majority of staff members and residents of mental health care facilities will have no experience in initiating health promoting interventions. Therefore, the project has to use a strategy of supported decision making, allowing staff members and residents to select the most appropriate health promoting actions. A strategy is needed which enables people in mental health care facilities to routinely identify the most relevant health problems, explore the main causes for these health problems, and then choose appropriate intervention programmes and systematically evaluate their effects.

## Competing interests

The authors declare that they have no competing interests.

## Authors' contributions

TB, RK, JH and SP have designed the HELPS project. PW, KA, LB, MD, ED, MM, TL, KPK, RL, PMJ, PP, SP, AG and JW are the national project coordinators. PW, RK, TB, CL, SP, RL and DMD have drafted the manuscript. All authors contributed and have approved the final manuscript.

## Pre-publication history

The pre-publication history for this paper can be accessed here:


